# Intraoperative Transesophageal Echocardiography Evaluation of Tricuspid Valve Re-repair for Ebstein’s Anomaly

**DOI:** 10.7759/cureus.46811

**Published:** 2023-10-10

**Authors:** Masashi Yoshida, Hisakatsu Ito, Mitsuaki Yamazaki

**Affiliations:** 1 Anesthesiology, Toyama University Hospital, Toyama, JPN; 2 Anesthesiology, Toyama Nishi General Hospital, Toyama, JPN

**Keywords:** cone reconstruction, tricuspid stenosis, tricuspid regurgitation, tricuspid valve, intraoperative transesophageal echocardiography, adult congenital heart disease, ebstein’s anomaly

## Abstract

Postoperative stenosis or regurgitation of the tricuspid valve is common and affects the prognosis after repair surgery of Ebstein’s anomaly. However, it is unclear how intraoperative echocardiography influences the postoperative course. We report a longitudinal echocardiography course including intraoperative transesophageal echocardiography in a cone reconstruction procedure for Ebstein's anomaly in a 17-year-old woman. Tight tricuspid valvuloplasty was preferred, but the tricuspid annulus enlarged rather after surgery. The evaluation of the tricuspid valve form and function using intraoperative echocardiography could support the surgeon’s impression.

## Introduction

Ebstein's anomaly is a congenital heart disease caused by a malformation of the tricuspid valve (TV), and its main manifestations are tricuspid regurgitation (TR) and atrialized right ventricle (RV) [1,2].

Surgical treatment varies from biventricular repair to single ventricle repair depending on TV morphology and RV function. Biventricular repair with an autologous valve includes the Danielson and Carpentier techniques, as well as cone reconstruction of the TV [1-3]. Postoperative complications in RV and TV function, especially residual TR and development of tricuspid stenosis (TS), are common issues affecting the prognosis of patients who underwent biventricular repair [4]. Biventricular repair with intraoperative evaluation of postplastic TV function can be expected to result in appropriate repair and prevent a second surgery. However, it is not clear how the findings of the intraoperative transesophageal echocardiography (TEE) influence the postoperative course. We report here the longitudinal echo course including intraoperative TEE in cone reconstruction of the TV for Ebstein's anomaly. Furthermore, we propose a reconstruction strategy of TV repair using intraoperative TEE evaluation for Ebstein's anomaly.

## Case presentation

The patient was a 17-year-old woman (height 149.5 cm, weight 42.5 kg, body surface area 1.30 m^2^) with a history of Ebstein’s anomaly. She had been diagnosed at birth with type C Ebstein's anomaly (according to the Carpentier classification), atrial septal defect (ASD), and TR.

At the age of 12 years, she began experiencing shortness of breath and fatigue during exercise that increasingly worsened over time. Pulse oximetry at rest was 95% (at room air). Catheterization revealed a right ventricular end-diastolic volume (RVEDV) of 182 mL (208.9% of normal values), a right ventricular ejection fraction (RVEF) of 43%, severe TR, right atrial enlargement, and right ventricular volume overload due to atrialized RV. She underwent biventricular repair via the Carpentier technique, which involved anterior leaflet mobilization and re-attachment to the annulus, and ASD closure. The level of residual TR immediately after surgery was mild, but it worsened to moderate-to-severe on postoperative day 21. No additional surgery was performed at this time. She was treated with angiotensin-converting enzyme inhibitors and diuretics, but as TR became severe and heart failure gradually worsened over five years, she was scheduled for re-repair of Ebstein’s anomaly via cone reconstruction of the TV.

At the time of the second surgery, her heart rate was 85 beats/min, her blood pressure was 95/57 mmHg, and her pulse oximetry was 98% (at room air). The degree of her heart failure was determined to be New York Heart Association (NYHA) class Ⅲ. Cardiomegaly was evident on a chest x-ray, and an electrocardiogram showed sinus rhythm, right bundle branch block, right axis deviation, QT prolongation, and negative T waves in leads Ⅱ, Ⅲ, and augmented vector of left foot (aVF). Preoperative transthoracic echocardiography (TTE) revealed an enlarged right atrium (RA) and RV, a right ventricular fractional area change (RVFAC) of 39.5%, a tricuspid annular plane systolic excursion (TAPSE) of 22.9 mm, an end-diastolic tricuspid valve diameter (TVD) of 37 mm, a TR peak velocity of 2.2 m/s, a TR peak pressure gradient (PG) of 19.36 mmHg, and wide regurgitation jet from the septal leaflet side. No TS was detected (Table 1). RV function was preserved, but TR and right atrial and ventricular enlargement were assessed to be worsening over time. Blood tests revealed that the only abnormal value was a mild elevation in the levels of N-terminal prohormone of brain natriuretic peptide (NT-proBNP) (70 pg/mL).

**Table 1:** Changes over time in echocardiographic findings. LVEF: left ventricle ejection fraction; NA: not available; N.p.: not particular; RVFAC: right ventricular fractional area change; TAPSE: tricuspid annular plane systolic excursion; TR: tricuspid regurgitation; TS: tricuspid stenosis; TVD: tricuspid valve diameter; PG: pressure gradient

Variables	Pre-op	Intra-op	14 days post-op	60 days post-op	Reference range
RVFAC (%)	39.5	39.5	NA	NA	47±7
TAPSE (mm)	22.9	16	NA	13	23±7
TVD (mm)	37	20.8	21.3	25.2	<40
TR	Severe	Slight	Mild	Mild	-
TS	N.p.	Mean PG=4	Mean PG=2	N.p.	Mean PG<2
LVEF	68	56	75	69	66±5

**Figure 1:**
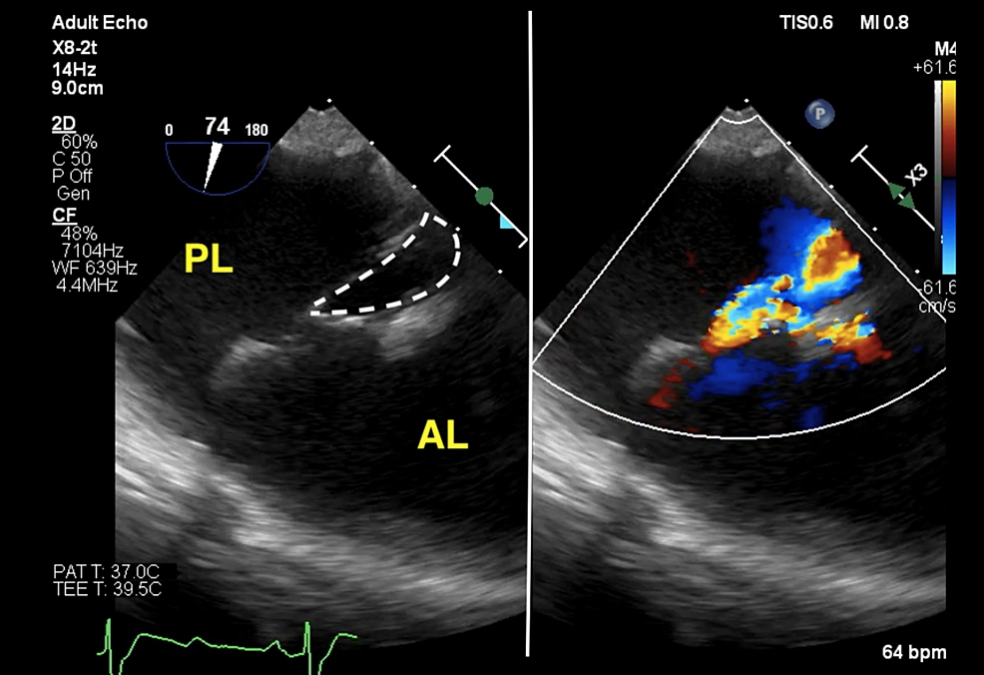
Transgastric TV short axis view from intraoperative TEE. Severe tricuspid regurgitation from the septal leaflet side, characteristic of Ebstein’s anomaly, can be observed. The dotted line indicates the area corresponding to septal leaflet defect. AL: anterior leaflet; PL: posterior leaflet; TEE: transesophageal echocardiography; TV: tricuspid valve

The patient received midazolam (5 mg) before being taken to the operating room, where an arterial catheter was inserted through the left radial artery. General anesthesia was induced with midazolam 3 mg, fentanyl 0.2 mg, and rocuronium 40 mg. The patient was ventilated with 100% oxygen, and after adequate muscle relaxation, the trachea was intubated and mechanical ventilation was initiated at a rate of 14 breaths per minute and a tidal volume of 370 mL. Maintenance of general anesthesia was accomplished via oxygen, fentanyl, rocuronium, and sevoflurane. A postintubation arterial blood gas analysis on FiO_2_ 0.4% revealed a PaO_2_ of 209.0 mmHg, a PaCO_2_ of 32.6 mmHg, a pH of 7.432, a level of HCO_3_- of 21.4, and a base excess of -1.7. After intubation, a central venous catheter was placed through the right internal jugular vein. The superior vena cava oxygen saturation was 72.6 mmHg. Intraoperative TEE before TV repair showed regurgitation from the septal leaflet side (Figure 1).

The anterior leaflet showed good mobility. The TR peak pressure gradient (PG) was 10 mmHg, the RVFAC was 40.3% and the hepatic venous blood flow waveform showed a systolic retrograde wave.

Anatomical observations of the TV revealed a large defect between the anterior and posterior leaflets. There was a gap between the septal leaflet and the posterior leaflet. The anterior leaflet was mobile. The original septal leaflet was attached to the right ventricular side and was not mobile. Cone reconstruction of the TV was performed using the anterior leaflet. Specifically, the valve ring was sutured, the anterior and posterior leaflet tissues were mobilized, and then the sides of all the mobilized leaflets were connected in a manner to create 360 degrees of leaflet tissue.

**Figure 2:**
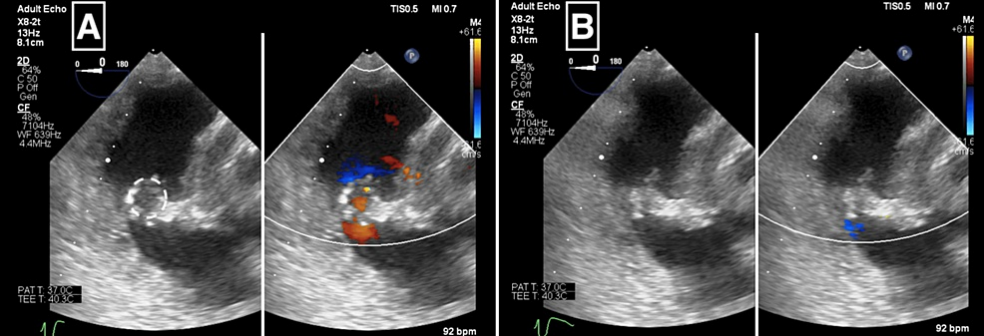
Transgastric TV short-axis view from intraoperative TEE after cone reconstruction of the TV. The images show diastole (A) and systole (B). The dotted line indicates the area corresponding to the TV orifice. TEE: transesophageal echocardiography; TV: tricuspid valve

**Figure 3:**
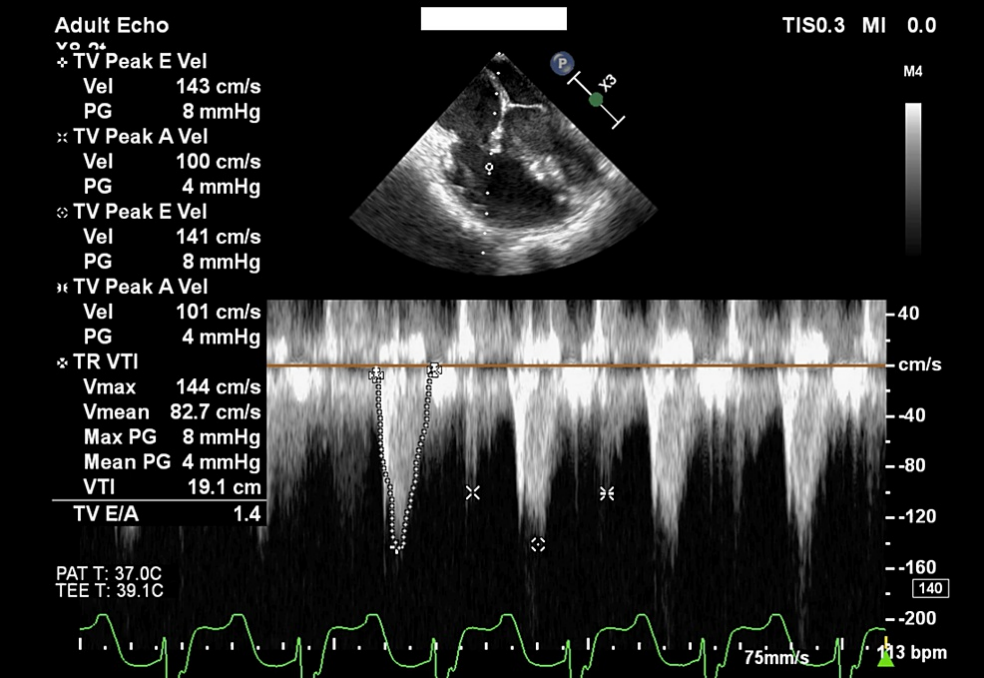
Four-chamber view from intraoperative TEE after cone reconstruction of the TV. TV mean PG was 4 mmHg and max PG was 8 mmHg. TEE: transesophageal echocardiography; TV: tricuspid valve; PG: pressure gradient

After TV repair, the patient was weaned from cardiopulmonary bypass (CPB) while receiving dopamine (2 μg/kg/min), dobutamine (2 μg/kg/min), nitroglycerin (1 μg/kg/min), and milrinone (0.2 μg/kg/min), and her own beat was in sinus rhythm. New arrhythmia was not observed. TV function was then evaluated via TEE. TV mean PG was 4 mmHg, max PG was 8 mmHg, the TVD was 20.8 mm, and a trace TR was evident (Figures 2A, 2B, 3). The blood pressure was 90/50 mmHg and the central venous pressure was 6 mmHg. TS was tolerated because of fears that therapeutic intervention could potentially exacerbate TR. Ninety minutes after withdrawal from CPB, TEE demonstrated a decreased left ventricle ejection fraction (LVEF), an E/e' index of 9.5, a kept RVFAC, and a decreased TAPSE (Table 1).

The total bypass time was 226 minutes, with an aortic cross-clamp time of 151 minutes. The total time under anesthesia was 587 minutes. The infusion volume was 1800 mL, the blood transfusion volume was 1630 mL, the blood loss was 580 mL, the urine volume was 820 mL, and the CPB volume loss was 802 mL. After the procedure, the patient was transferred to the intensive care unit under intubation and was administered dopamine (3 μg/kg/min), dobutamine (3 μg/kg/min), noradrenaline (0.02 μg/kg/min), nitroglycerin (0.2 μg/kg/min), and milrinone (0.2 μg/kg/min). The postoperative course was uneventful, and she was extubated the following day.

On postoperative day 14, TTE showed an increased TVD, mild TR, and mild TS (TV mean PG was decreased). Left ventricular contractility was maintained at an LVEF. The patient was discharged on that day. TTE performed two months after the surgery showed an increased TVD, mild TR, no TS, a decreased TAPSE, and a well-kept LVEF (Table 1). She continues to be monitored via outpatient follow-up.

## Discussion

RVFAC and TAPSE are parameters commonly used for the evaluation of RV function via echocardiography. For the evaluation of TV function, the severity of TR is determined from the effective regurgitant orifice area (EROA), the regurgitant volume, the regurgitation jet area, and the TVD, whereas the severity of TS is determined from mean PG and valve opening area [5]. Each of these evaluations of TV morphology and RV function by preoperative echocardiography is useful for determining the therapeutic strategy and prognosis in cases of Ebstein's anomaly [4,6]. Booker and Nanda reported that a large detachment of the anterior leaflet favors TV repair [6]. In this particular case, the patient had already undergone surgical repair via the Carpentier technique, and the treatment options were therefore either TV repair or replacement. Based on the results from the preoperative TTE, which showed sufficient size and mobility of the anterior leaflet, we determined that TV repair was suitable for this patient. Although several reports have mentioned the importance of intraoperative TEE in the evaluation of TV and RV function, there have been no reports describing actual measurements of intraoperative TEE and postoperative follow-up for TV repair in cases of Ebstein's anomaly [7-9].

The relationship between these TEE findings and prognosis is still unclear. In the present case, RV function (evaluated through RVFAC and TAPSE after the patient was weaned from CPB) was preserved. Although we recognized mild TS and slight TR, we did not perform additional interventions to correct the TS after taking into account the mean PG, the low central venous pressure value, and the possibility of postoperative TR exacerbation.

Up until two months after surgery, TVD tended to become enlarged and TAPSE was reduced, TS improved, but TR became worse compared with intraoperative findings (Table 1). This case also suggests that the pressure gradient in TV assessed by intraoperative TEE can potentially improve within a few months. Therefore, it could be better to perform TV plasty with a narrow orifice area. However, it's not clear whether all patients undergoing cone reconstruction of the TV will follow the same course. It is also necessary to investigate what levels of TS and TR and what degree of reduction in RV function are acceptable in a cohort study.

Brown et al. reported that the 20-year survival for TV repair was 76%, and survival free from reoperation at 20 years for valve repair was 53% for patients operated on at less than 12 years of age and 57% for patients operated on at more than 12 years of age [4]. Brown et al. reported that preoperative deterioration of RV function is associated with increased postoperative mortality and reoperation rates [4]. In this case, the preoperative RV function was preserved, RVFAC was 39.5% and TAPSE was 22.9 mm. The appropriate time for surgery is therefore when the RV function has decreased over time, but is still maintained.

Postoperative evaluation of RV function is also useful for prognosis purposes. RV function after cone reconstruction of the TV for Ebstein's anomaly decreases in the first few years after surgery [10]. A decline in RV function in the postoperative course was indeed observed in this case, which can be explained by the increase in RV afterload and its consequences. However, further observations are required to see whether RVFAC and TAPSE can adequately assess right ventricular function in the preoperative, intraoperative, and postoperative status of Ebstein's anomaly.

In addition, Brown et al. reported that postoperative mortality and reoperation rates increased with moderate-to-severe LV systolic dysfunction in TV repair for Ebstein's anomaly [6]. TV function and LVEF were evaluated in our patient, and LV function was relatively well maintained (Table 1).

## Conclusions

The function of the repaired TV was evaluated intraoperatively by TEE during cone reconstruction of the TV in a patient affected by Ebstein's anomaly. Intraoperative TS was improved in the postoperative course, while TR increased slightly. This suggests that mild TS during intraoperative TEE evaluation is acceptable and tight valvuloplasty could be preferable. TEE immediately after TV repair is useful for evaluating TV function, especially the degree of TS, as it is difficult to assess by visual inspection by the surgeon. Additional data on intraoperative TEE and its association with prognosis is required for the appropriate evaluation of RV function and postplastic TV function in surgery for Ebstein's anomaly.
